# Research trends in oral health and frailty studies: a bibliometric and visual analysis

**DOI:** 10.3389/fmed.2025.1610582

**Published:** 2026-01-02

**Authors:** Shiqi Chen, Feng Tian, Ping-Ping Huang, Rongxiang Zhang, Chenyang Zhu, Yuan Chen

**Affiliations:** 1 Fujian Branch of National Clinical Research Center for Cardiovascular Diseases, School of Medicine, Xiamen Cardiovascular Hospital of Xiamen University, Xiamen, China; 2 School of Nursing, Fujian University of Traditional Chinese Medicine, Fuzhou, China

**Keywords:** bibliometrics, scientometric analysis, oral health, frailty, aging

## Abstract

**Objectives:**

As the global population ages, the association between oral health and frailty has garnered increasing attention. Declining oral function is closely linked to frailty syndromes. This study employs bibliometric methods to systematically analyze research hotspots and emerging trends in this field.

**Methods:**

A comprehensive bibliometric analysis was conducted using data from the Web of Science Core Collection (2000–2024) and visualized with CiteSpace software. Key metrics included annual publication trends, contributions by countries/institutions and authors, journal influence, co-citation networks, and keyword clustering related to oral health and frailty.

**Results:**

A total of 270 publications were analyzed. The research landscape was characterized by decentralized contributions. At the country level, Japan was the leading contributor, followed by China and the United States. Institutionally, the University of London and several Japanese institutions were the most productive. While Professor Hirohiko Hirano emerged as the most prolific and cited individual author, this productivity was not supported by a dominant, large-scale collaborative network, indicating a fragmented authorship structure. Gerodontology published the most studies, while the Journal of the American Geriatrics Society exerted the strongest academic influence. Key research themes centered on tooth loss as a pivotal risk factor, alongside the association between oral health and frailty, and its impact on quality of life.

**Conclusion:**

Despite a rapidly growing body of research on the link between oral health and frailty, international collaboration in this field remains limited. The bibliometric analysis reveals a robust and well-established research domain supporting the association between oral conditions and frailty, as evidenced by dense co-citation networks and coherent keyword clustering, with implications for cognitive decline and reduced quality of life. Current assessment tools lack standardization, and future studies should explore the synergistic effects of oral health interventions alongside conventional frailty management strategies.

## 1 Introduction

Global population aging has underscored the importance of healthy aging. Oral health, a vital component of overall wellbeing, encompasses essential functions such as mastication and swallowing (1). Emerging evidence highlights the significant role of poor oral health in systemic health decline, garnering increasing scientific interest. In older adults, oral health is directly linked to general health outcomes (2).

Frailty, an age-related syndrome characterized by diminished physiological reserve, has become a major public health concern. Its prevalence increases with population aging (3) and is strongly associated with physical decline, disability, chronic diseases, hospitalization, and mortality (4, 5). Recent studies have established specific oral health factors—such as tooth loss, masticatory efficiency, and salivary function—as modifiable correlates of frailty (2, 6, 7). For instance, Kamdem et al. demonstrated that impaired masticatory function is a significant predictor of frailty (8), while Tanaka et al. identified poor oral health, assessed through measures of masticatory function, tongue pressure, and salivation, as an independent risk factor (9). Furthermore, oral health is intrinsically linked to nutritional status. Tooth loss or diminished chewing capacity can lead to malnutrition, thereby fostering the development of frailty (10). The underlying mechanisms connecting oral health to frailty are multifaceted, encompassing pathways such as tooth loss, hyposalivation, periodontal disease, and dental caries (11). Consequently, oral health constitutes not only a vital determinant of quality of life among older adults but also a critical nexus for multidisciplinary health management.

Nurses play a pivotal role in assessing and managing oral health in frail older adults, as they are often the first to identify early signs of oral dysfunction during routine care (12). Poor oral health exacerbates frailty by compromising nutrition, social engagement, and systemic health—key domains where nursing interventions are essential. Despite extensive research on this relationship, the field lacks a systematic synthesis of its knowledge structure and evolving trends. Bibliometric analysis, leveraging information visualization tools like CiteSpace, offers a robust solution. This Java-based application employs co-citation, co-occurrence, and clustering analyses to map disciplinary landscapes, revealing research hotspots and intellectual networks (13). As a quantitative method, bibliometrics enables objective tracking of scientific progress through statistical and visual techniques (14), widely applied in medical research. This study employs CiteSpace to analyze the research trajectory in oral health and frailty, identifying key themes and future directions.

## 2 Materials and methods

### 2.1 Data source

The Web of Science (WoS) Core Collection was selected for its rigorous journal curation (based on Bradford’s law) and widespread adoption in bibliometric studies (15).

### 2.2 Search strategy

A systematic literature search was conducted in the Web of Science Core Collection on 16 January 2025 to identify relevant publications on the association between oral health and frailty. The search strategy was constructed using comprehensive sets of terms related to “oral health” (e.g., Mouth Diseases, oral hygiene, tooth loss) and “frailty” (e.g., frailty syndrome, debilitation). The search was limited to English-language articles and reviews published between 2000 and 2024. The full Boolean query and detailed search strategy are provided in Supplementary Material 1.

### 2.3 Inclusion and exclusion criteria

Inclusion criteria: original articles or reviews published in peer-reviewed journals. Through a combination of manual screening and the “delete duplicates” function provided by CiteSpace 6.3.1, we excluded ineligible documents based on the following exclusion criteria: (1) errata; (2) duplicate publications; (3) irrelevant documents.

### 2.4 Bibliometrics and visualization analysis

We exported the retrieved articles in plain text format with complete records and references named “download_XXX.txt” and then imported them into CiteSpace 6.3.1 for further analysis. In mapping the visual knowledge graph window, we followed the main procedural steps of CiteSpace, including time slicing, thresholding, modeling, pruning, merging and mapping (13). Nodes in different maps represent authors, institutions, countries, or keywords. The node size indicates the frequency of occurrence or citation and the color of the node indicates the year of occurrence or citation. In addition, nodes with purple pruning indicate higher mediator centrality. Centrality reflects the role of a node in a knowledge network, and nodes with high intermediate centrality (> 0.1) are usually considered as turning points or key points in a domain (14, 16). Links between nodes indicate co-occurrence relationships, and the thickness of the links indicates the strength of co-occurrence; the more links there are, the thicker and tighter the relationship between nodes. The specific parameters configured in CiteSpace for the construction and pruning of each network type are detailed in Supplementary Material 2.

## 3 Results

### 3.1 Publication trends

**FIGURE 1:**
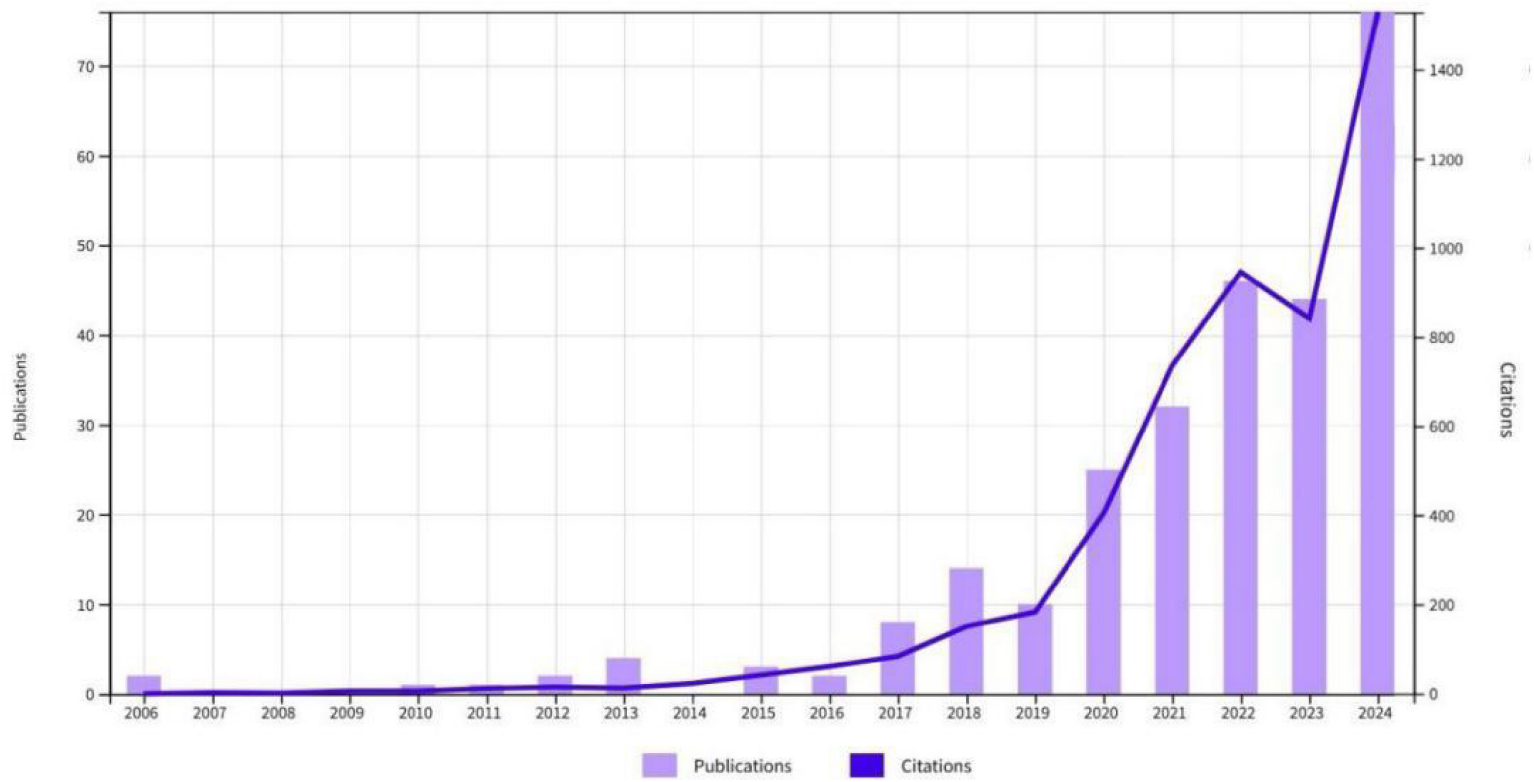
Annual publication volume and citations.

Analysis of the 270 included publications revealed a clear upward trajectory in annual output (Figure 1). Although no relevant studies were published before 2006, there was notable growth between 2019 and 2022, culminating in a peak of 76 publications in 2024. The observed temporary declines in 2019 and 2023 did not alter the overall upward trend. This surge aligns with global demographic shifts and the heightened focus on aging-related health, particularly the role of oral health in frailty. The expansion is likely driven by increased research investment and interdisciplinary collaboration.

### 3.2 Countries and institutions analysis

**FIGURE 2:**
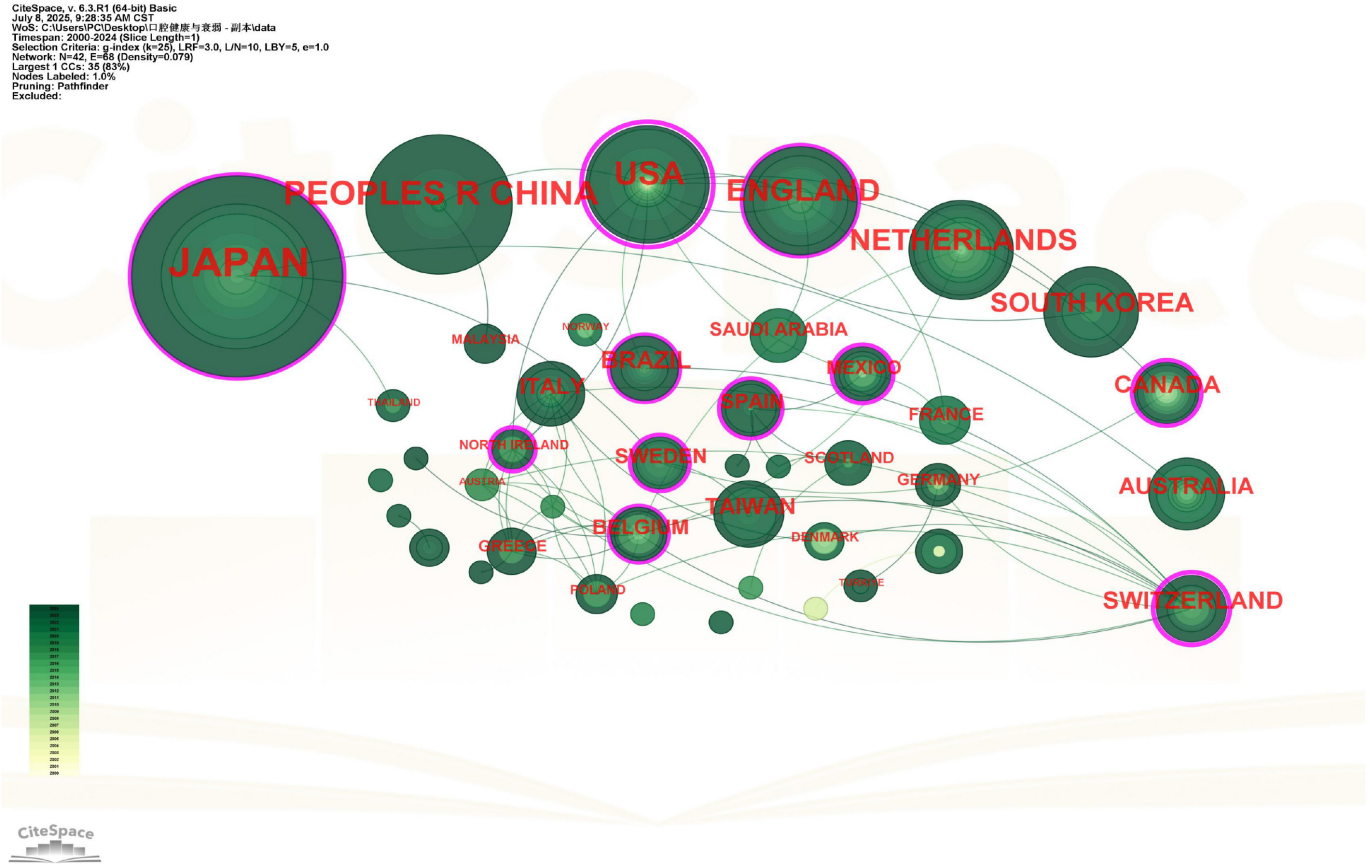
Viewer’s visualization map of countries and regions. Japan has the highest number of published papers. Nodes represent countries/regions, sized by publication volume and colored by the average publication year (yellow for earlier, green for more recent publications). Connecting lines indicate collaborative relationships, with thickness denoting strength. Nodes with purple rings are pivotal hubs (betweenness centrality ≥ 0.1). Japan, the largest node, led in publication output.

A total of 42 countries contributed to research on oral health and frailty, forming 68 collaborative networks (Figure 2). The top 10 contributing countries alone accumulated 5,316 citations (see Supplementary Table 1), corresponding to an average of approximately 21 citations per publication within this group. Japan was the most productive nation, contributing 83 publications (approximately one-third of the total) and accumulating the highest citation count of 2,295. Its average of approximately 28 citations per publication reflects a substantial output and influence, underscoring the country’s strong research focus and significant impact in this domain. This leadership position likely stems from Japan’s rapidly aging population (17) and its advanced research infrastructure at the intersection of dental science and geriatric medicine (18). China (*n* = 36) and the United States (*n* = 30) ranked second and third in publication output. Notably, Japan, the United States, the United Kingdom, and Switzerland demonstrated the highest betweenness centrality scores (> 0.1), indicating their pivotal roles as knowledge hubs in this research field (see Supplementary Table 1). The resulting network density of 0.079 indicates limited and fragmented international cooperation. Japan not only emerged as the most productive nation but also served as a central hub in the collaboration network, with direct links to 12 other countries. The United States demonstrated the highest betweenness centrality (0.46), functioning as the most critical bridge for international knowledge exchange. European nations, particularly England and Switzerland, formed a distinct collaborative cluster, while Asian countries showed relatively sparse cross-regional partnerships.

**FIGURE 3:**
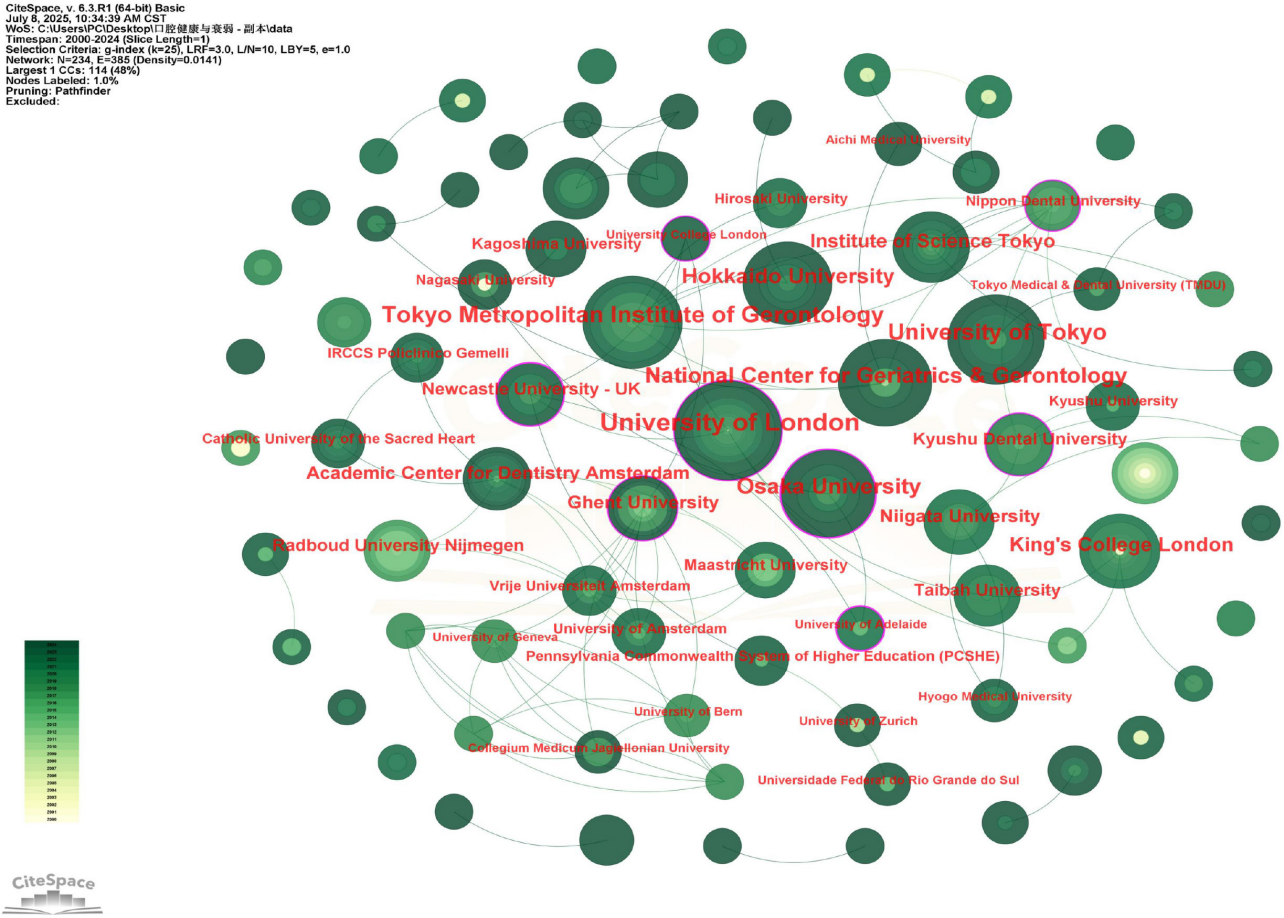
Viewer’s visualization map of organizations. Mainly focused on Japanese and British issuing organizations. Nodes represent research institutions, with size proportional to publication count and color indicating the average publication year (yellow for earlier, green for recent). Lines denote collaborative ties. Key bridging institutions are highlighted with purple rings. The network is dominated by Japanese and British institutions.

At the institutional level, organizations from the UK and Japan dominated the research landscape (Figure 3). The University of London led with 17 publications, followed by the Tokyo Metropolitan Institute of Gerontology (*n* = 14) and the University of Tokyo (*n* = 13). Interestingly, while the University of Newcastle, University of Adelaide, and University College London published fewer articles (not ranking in the top 10 by volume), they exhibited high centrality scores, suggesting their disproportionate influence as bridging nodes in the research network (see Supplementary Table 2). The institutional collaboration network exhibited a much lower density of 0.0141 (Figure 3), quantitatively confirming that research partnerships are highly concentrated and have not yet formed a widely interconnected network. Together, these network metrics (country density: 0.078; institutional density: 0.0141) quantify a clear trend: research collaboration in this field, while growing, remains concentrated around a few highly productive nations and institutions, rather than being a pervasive, worldwide network.

### 3.3 Authors and co-cited authors analysis

**FIGURE 4:**
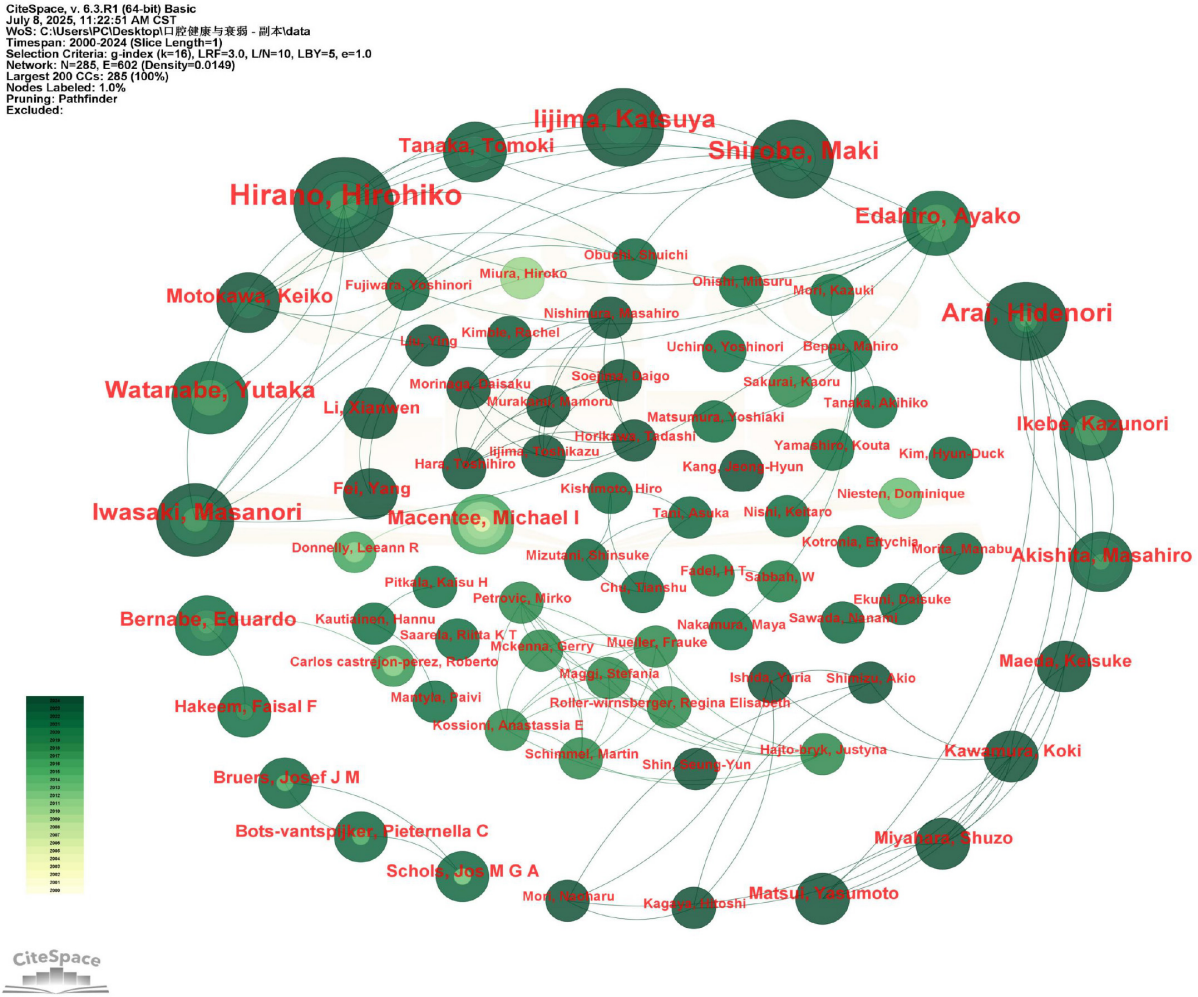
Viewer’s visualization map of authors. Hirohiko Hirano was the most published author in this field. Nodes represent authors, scaled by their number of publications and colored by the average publication year (yellow for earlier, green for recent). Co-authorship is shown by connecting lines. Authors with purple rings have high betweenness centrality. Hirohiko Hirano, represented by the largest node, was the most prolific author.

A total of 297 distinct authors were identified as contributors to the 270 publications analyzed (Figure 4). The top 10 authors, ranked by number of publications, were collectively cited 5,816 times, with an average of approximately 582 citations per author. Professor Hirohiko Hirano emerged as the most influential scholar, with 10 publications that accumulated 1,288 citations. Notably, his work achieved an average of approximately 129 citations per publication, a rate that substantially exceeds the group average and underscores his pivotal influence in the field. His seminal work has been particularly impactful in three key areas: (1) conceptualizing oral frailty, (2) developing standardized assessment tools, and (3) exploring innovative intervention strategies. Among the remaining top 10 authors, publication counts showed minimal variation (see Supplementary Table 3), suggesting the absence of a dominant research group. A key trend observed in authorship is its structural fragmentation. This observation is quantitatively supported by the author collaboration network density of 0.0149 and the co-cited author network density of 0.0154, both of which are very low. These values confirm the fragmentation of the research community and the lack of a tightly-knit collaborative group or a dominant intellectual school. While this fragmentation may reflect the interdisciplinary nature of oral frailty research, it also suggests the field would benefit from more coordinated collaborative networks to advance consensus and standardization.

### 3.4 Journal and cited journal analysis

**FIGURE 5:**
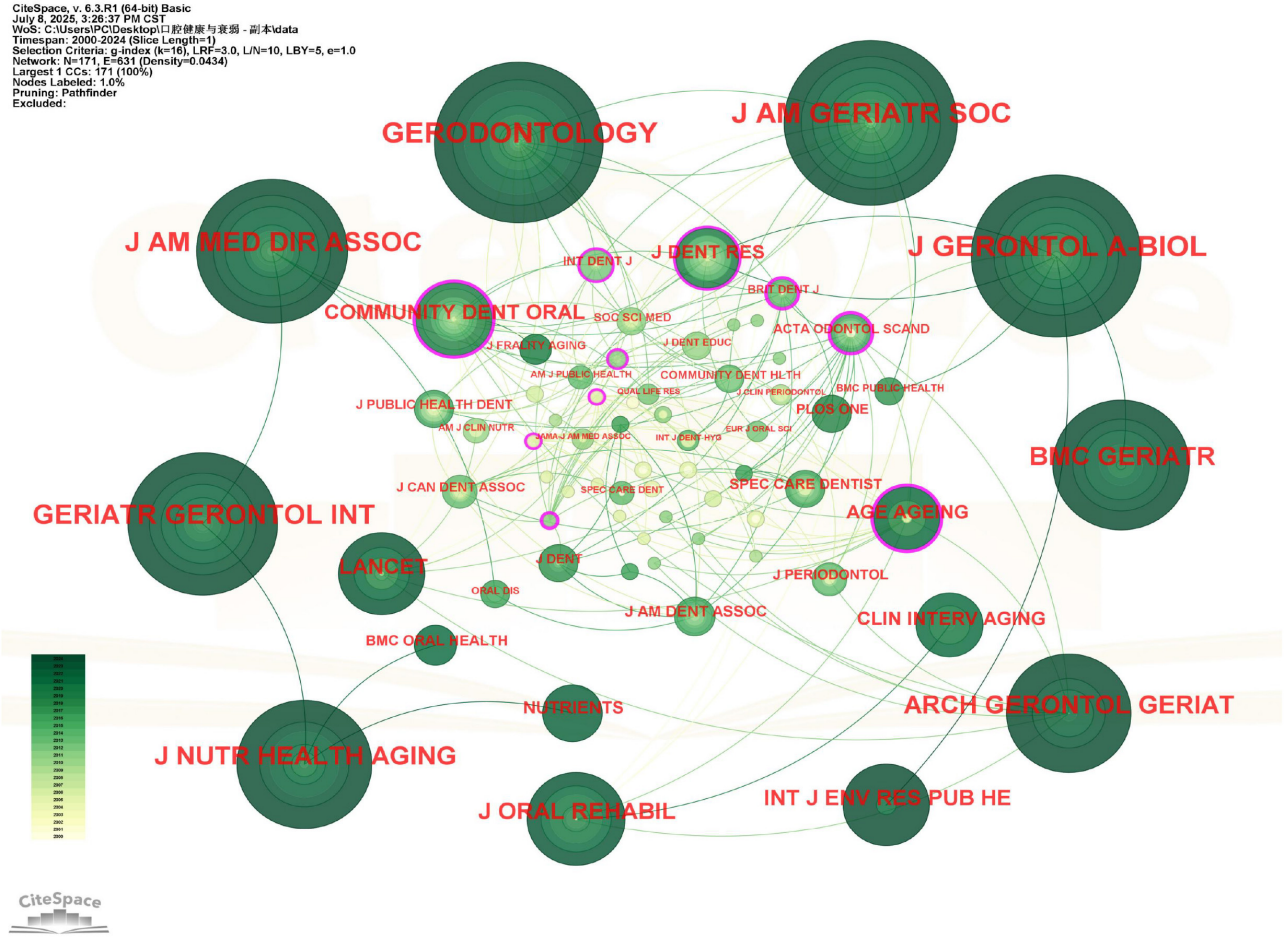
Viewer’s visualization map of journals. Gerodontology was the most published journal in this field. Nodes represent journals, sized by their co-citation frequency and colored by the average year of citation (yellow for earlier, green for recent). Lines represent co-citation relationships. Journals with purple rings are central to the knowledge structure. Gerodontology was the most frequently co-cited journal.

The 270 publications were disseminated across more than 85 academic journals (Figure 5). The top 10 journals published 143 articles, which received 3,008 citations, resulting in an average of approximately 21 citations per article. The Journal of the American Geriatrics Society demonstrated the highest impact per publication among these, with an average of 71 citations per article. Gerodontology emerged as the most prolific journal, publishing 20 articles (7.4% of total publications) on this topic (see Supplementary Table 4), underscoring its role as a key platform for oral health and frailty research. The journal co-citation network density was 0.0434, meaning that researchers in this field tend to cite a common, albeit not monolithic, set of core journals. Notably, while the Journal of the American Geriatrics Society published only 8 articles, it achieved a remarkable citation count of 568 (see Supplementary Table 4), demonstrating its substantial impact in this field. The journal’s publications addressed critical aspects of the oral health-frailty relationship, including mechanistic investigations and intervention efficacy assessments, providing valuable theoretical and practical guidance for subsequent research.

### 3.5 Co-citation analysis

**FIGURE 6:**
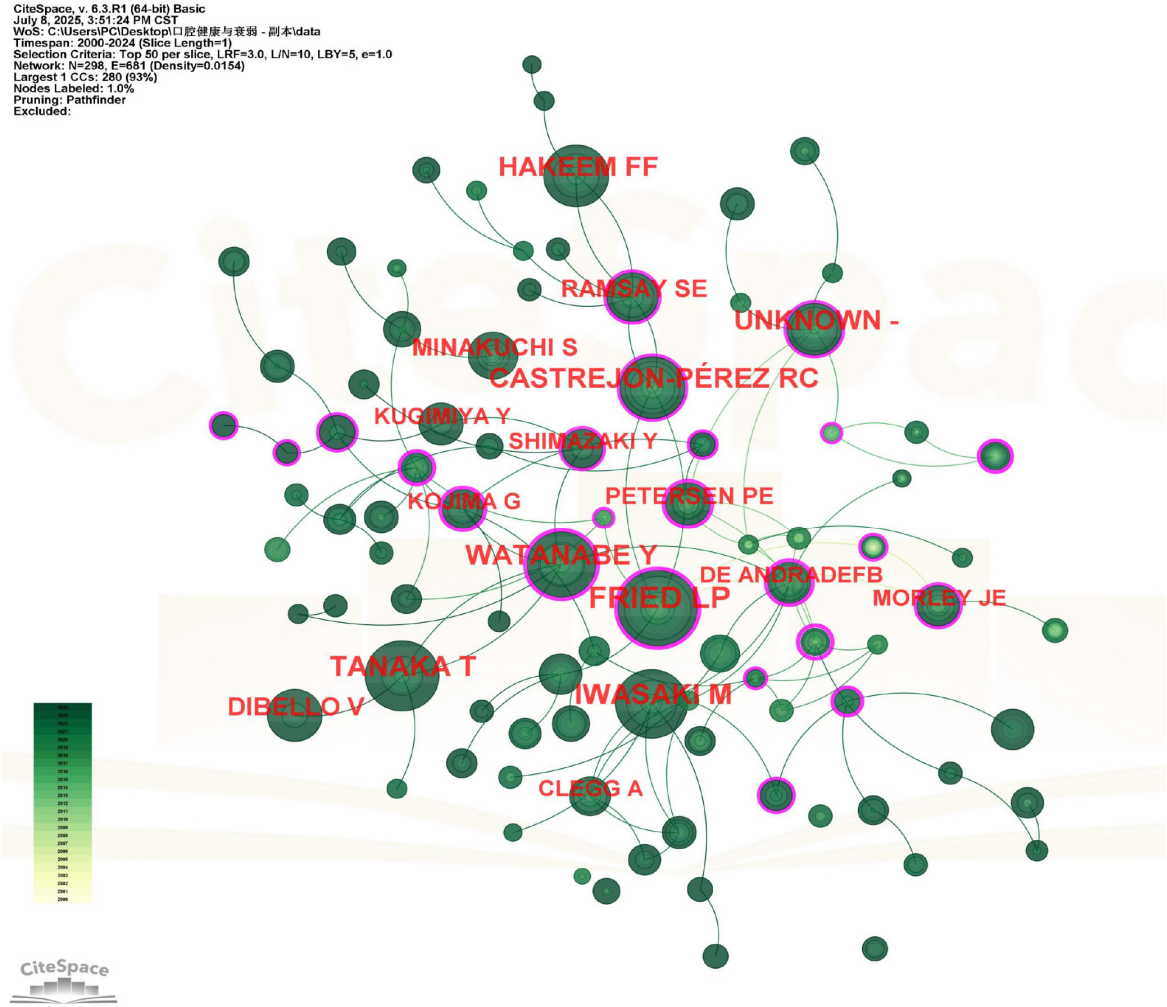
Viewer’s visualization map of Co-citation. Nodes represent cited references, sized by co-citation frequency and colored by the average citation year (yellow for earlier, green for recent). Co-citation links are shown between references. Landmark publications with high influence are highlighted by purple rings.

Co-citation relationships occur when two or more publications are cited together by subsequent works, indicating conceptual connections in the academic literature. Our analysis generated a co-citation network comprising 295 reference nodes (Figure 6), where node size corresponds to citation frequency and connecting lines represent co-citation relationships. The co-citation frequency served as our primary analytical metric, as it objectively measures a publication’s academic influence within a research domain. The five most frequently co-cited studies (see Supplementary Table 5) predominantly examined: (1) risk factors associated with oral health decline and frailty, and (2) mechanistic relationships between oral dysfunction and physical frailty in aging populations. The five most co-cited references were cited 206 times in total, averaging approximately 41 co-citations per study. This high frequency highlights their foundational role and widespread recognition as key literature in the field.

### 3.6 Keyword analysis

**FIGURE 7:**
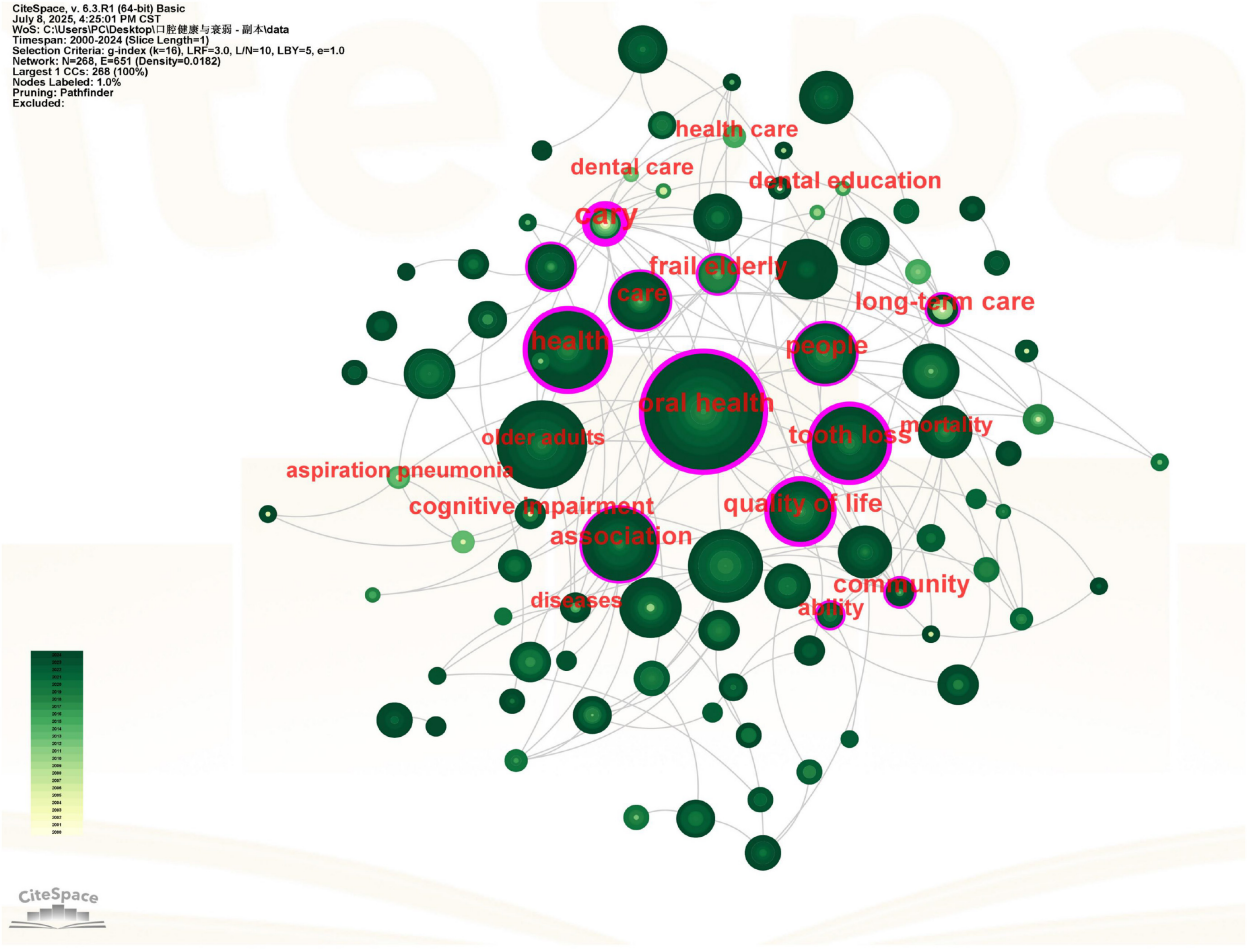
Viewer’s visualization map of keywords. Nodes represent keywords, sized by frequency of occurrence and colored by the average publication year of citing articles (yellow for earlier, green for recent). Lines indicate co-occurrence. Keywords with purple rings (e.g., “tooth loss”) function as conceptual bridges with high betweenness centrality.

A total of 157 keywords were analyzed to identify research hotspots and trends (19) (Figure 7). The network’s low density (0.0182) indicates a research landscape composed of several distinct, internally cohesive thematic clusters that have not yet coalesced into a tightly integrated framework. The most prominent keywords, based on frequency of occurrence and betweenness centrality, are summarized as follows. The top 10 keywords by frequency, along with their corresponding frequency and centrality values, were: “oral health” (Count = 123, Centrality = 0.42), “older adults” (Count = 60, Centrality = 0.05), “health” (Count = 53, Centrality = 0.41), “association” (Count = 47, Centrality = 0.36), “adults” (Count = 47, Centrality = 0.12), “tooth loss” (Count = 44, Centrality = 0.60), “people” (Count = 35, Centrality = 0.22), “quality of life” (Count = 33, Centrality = 0.20), “prevalence” (Count = 27, Centrality = 0.05), and “frailty” (Count = 26, Centrality = 0.04). Among these, “tooth loss” demonstrated the highest centrality value (0.60), highlighting its critical function as a conceptual bridge within the research network, despite not being the most frequently occurring term. Other keywords with notably high centrality included “oral health” (Centrality = 0.42) and “health” (Centrality = 0.41). The keywords related to oral health and frailty are: elderly population, quality of life, tooth loss, oral frailty and care. The hot keywords for the research direction were association and risk. Notably, “tooth loss” and “oral health-frailty association” demonstrated particularly high betweenness centrality scores (see Supplementary Table 6), suggesting their pivotal role as conceptual bridges in this research network.

**FIGURE 8:**
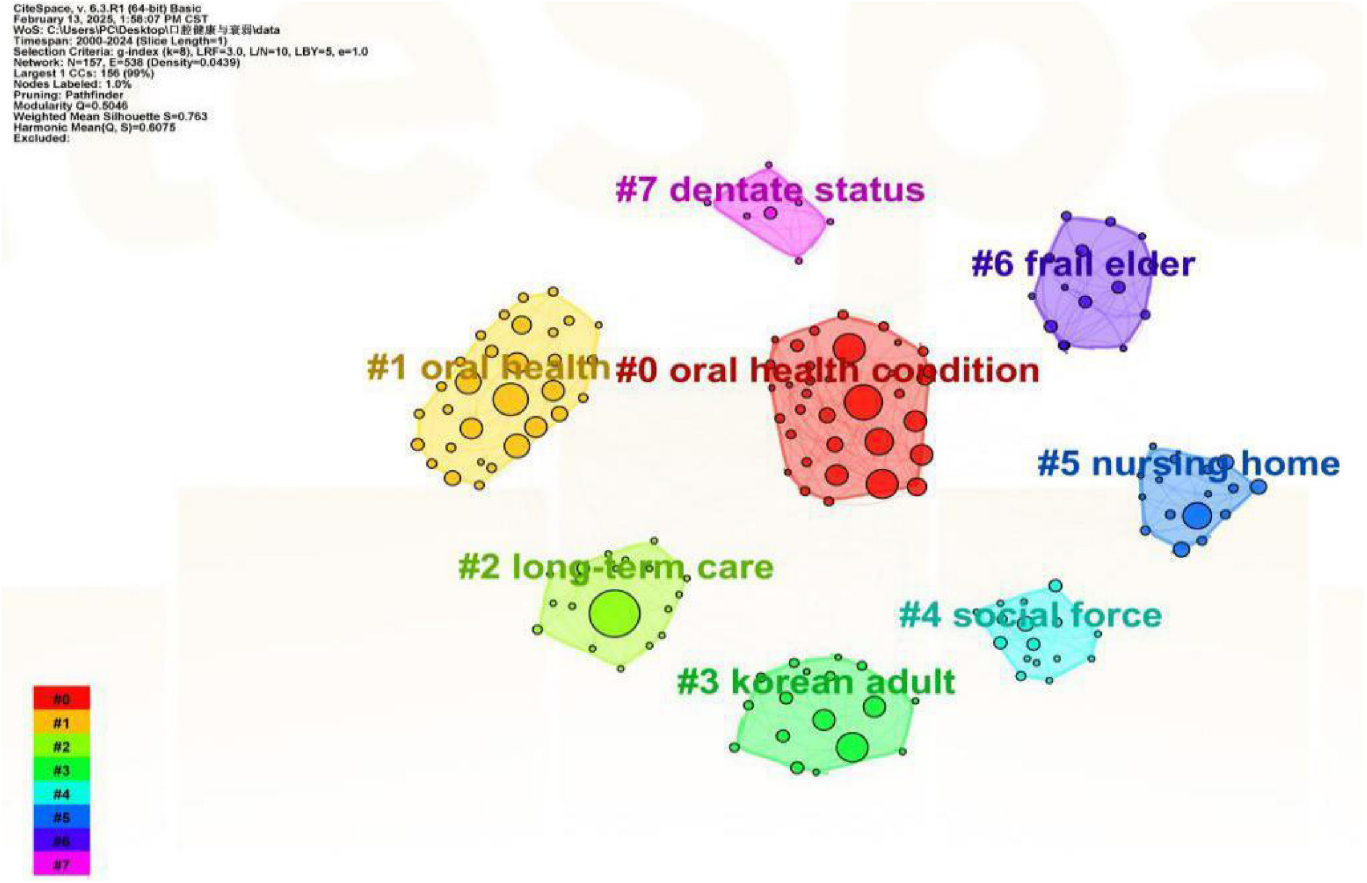
Co-occurring keyword clustering knowledge map. Clusters of closely related keywords, each assigned a unique color and automated label (e.g., #0, #1), represent distinct research themes. All clusters achieved silhouette scores > 0.7, confirming robust grouping. The map illustrates the intellectual structure of the field.

The clustering analysis yielded eight distinct thematic groups using log-likelihood ratio (LLR) statistics (Figure 8). The robustness of our clustering results was confirmed by silhouette coefficients exceeding 0.7 for all clusters (20, 21), indicating both methodological soundness and substantive meaningfulness of the identified research themes.

### 3.7 Analysis of keyword citation bursts

**FIGURE 9:**
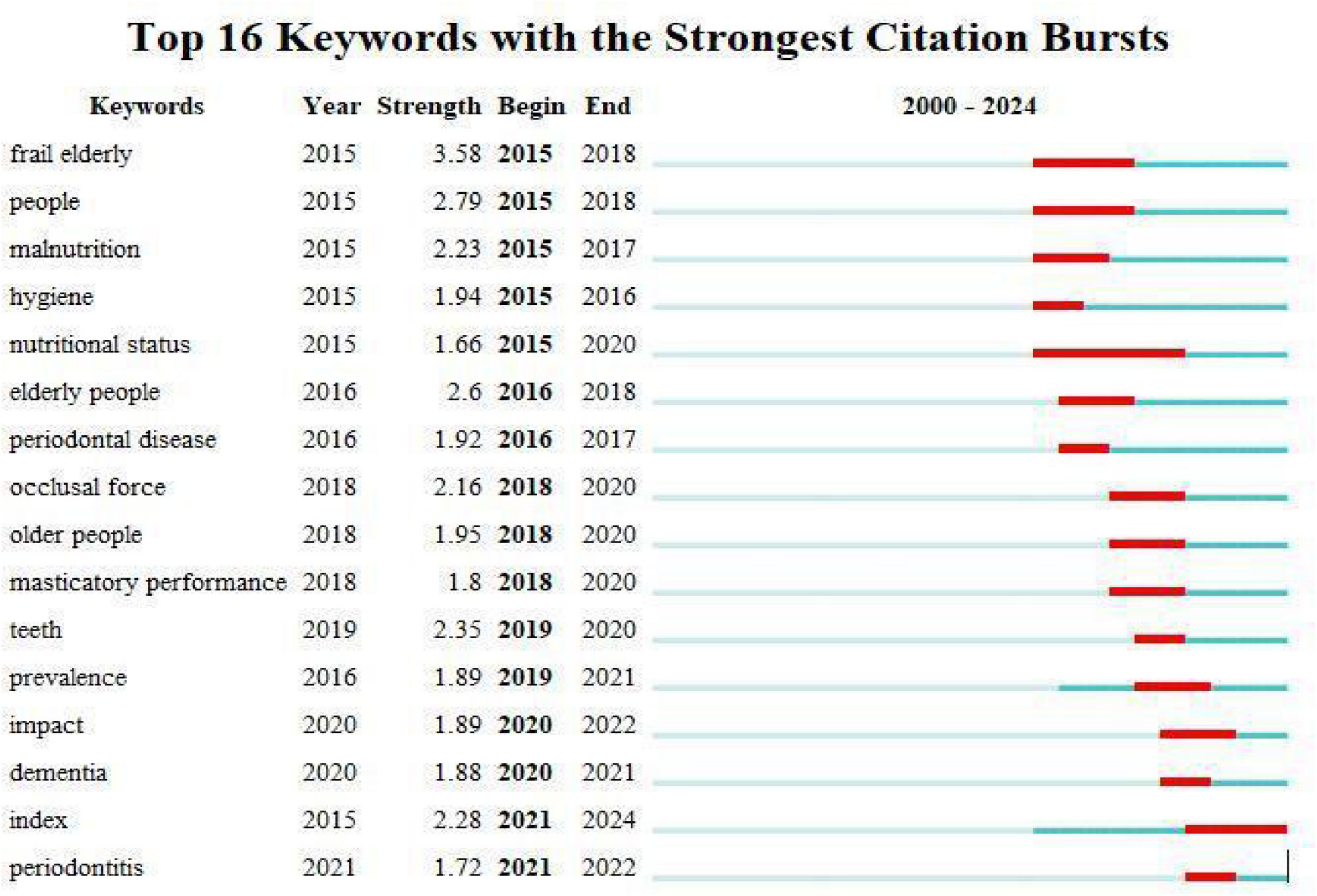
The top 16 keywords with the strongest citation bursts. Keywords exhibiting the most significant temporal surges in citation frequency are shown. The grey bar represents the study period (2000–2024); red segments indicate burst duration. “Strength” quantifies burst intensity; “Begin” and “End” define the burst period.

The temporal evolution of research trends in oral health and frailty was examined through a citation burst analysis of the 16 most prominent keywords from 2000 to 2024 (Figure 9). In the generated visualization, the blue timeline represents the entire study period, while red segments highlight intervals during which specific keywords experienced statistically significant surges in citation frequency, defined as a threefold or greater increase relative to baseline levels (21). This analytical approach provides valuable insights into shifting research paradigms, as emerging keywords with strong citation bursts effectively map the migration of scientific frontiers. Recent burst keywords are particularly indicative of current research hotspots, revealing a clear trajectory of conceptual advancement in the field. The analysis demonstrates a progressive refinement of research focus, evolving from the initial epidemiological characterization of frailty in elderly populations, through investigations of specific oral function parameters, and ultimately to the development and validation of standardized assessment tools. This evolution reflects both the maturation of the field and an increasing recognition of oral health as a critical determinant of frailty syndromes, with contemporary research emphasizing quantifiable clinical indicators and their predictive value for adverse health outcomes in aging populations.

## 4 Discussion

### 4.1 Advances in research on oral health and frailty

The marked growth in research output from 2000 to 2024, as evidenced by our bibliometric analysis, corroborates a robust association between oral health status and the development of frailty. This expanding body of literature reflects the growing scientific recognition of oral health as a critical determinant of geriatric health outcomes. Three nations—Japan, China, and the United States—have emerged as predominant contributors, collectively accounting for the majority of published works and significantly advancing the field’s theoretical and methodological foundations. However, our co-authorship network analysis revealed a notable deficiency in international research collaboration, characterized by limited cross-border partnerships among investigators. This finding is quantitatively underscored by the low network densities at the author (0.0149), institutional (0.0141), and even country (0.079) levels. These values confirm that the field is characterized by fragmented and decentralized research efforts, rather than strong, interconnected collaborative networks. Collectively, these findings underscore the need for enhanced multinational cooperation to facilitate knowledge exchange and comparative studies across diverse populations. Current research efforts primarily focus on three interconnected domains: (1) elucidating the pathophysiological mechanisms linking specific oral health conditions to frailty progression, (2) examining the clinical consequences of oral health-related frailty across functional domains, and (3) developing and validating standardized assessment tools capable of capturing the oral health-frailty continuum with greater precision.

### 4.2 Association between oral health and frailty

#### 4.2.1 The edentulous jaw and frailty

Keyword analyses converge to position “tooth loss” (Count = 44, Centrality = 0.60) not only as the conceptual core of the research domain but also as a pivotal bridge linking its disparate themes. This role, highlighted by its exceptional centrality, is validated by the frequent co-citation of seminal longitudinal studies that have established tooth loss as a central and independent risk factor for frailty. Emerging evidence from geriatric cardiovascular research establishes dentition status as a significant and modifiable predictor of frailty development, with particular implications for cardiovascular disease progression. The number of remaining teeth demonstrates a strong inverse correlation with frailty severity, where complete tooth loss (edentulism) is associated with markedly reduced maximum bite force and impaired masticatory efficiency (22, 23). Importantly, a threshold effect appears at approximately 20 teeth, with individuals maintaining this dentition level showing a 42% lower frailty prevalence compared to edentulous counterparts in adjusted analyses (24). This association remains robust across study designs, with systematic reviews of 17 prospective cohorts demonstrating a pooled hazard ratio of 1.81 for frailty incidence following tooth loss (25–27).

#### 4.2.2 Periodontal disease and frailty

The analysis of keyword citation bursts identified the successive emergence of “periodontal disease” and “periodontitis” as distinct research fronts, signaling a conceptual shift toward investigating advanced disease stages. An initial burst of “periodontal disease” (2016–2017) was followed by a later burst of the more severe “periodontitis” (2021–2022). This sequential pattern suggests a conceptual shift within the literature toward investigating advanced disease stages. The recurrence of this theme as an emerging frontier underscores its sustained elevance. Accumulating epidemiological evidence demonstrates a significant association between periodontal inflammation and frailty syndrome. A population-based cross-sectional investigation revealed that older adults with moderate-to-severe periodontitis exhibited 45% greater odds of frailty compared to those with a healthy periodontium (28). This association appears temporally meaningful, as longitudinal data indicate accelerated frailty progression among periodontitis patients relative to periodontally healthy controls (29). This relationship may be bidirectional, as frailty-associated physical limitations frequently compromise oral hygiene practices and dental care utilization among older adults (30). Mobility impairments reduce access to professional dental services, while decreased manual dexterity and fatigue often lead to inadequate plaque control, thereby exacerbating periodontal destruction and perpetuating a deleterious cycle of oral-systemic health decline.

#### 4.2.3 Oral sarcopenia in frailty pathophysiology

The bibliometric analysis identifies “oral frailty” as a consolidated research theme (see Supplementary Table 6), with its functional dimensions highlighted by citation bursts for “masticatory performance” and “occlusal force” (Figure 9). This pattern underscores a research consensus focusing on the decline of oral muscle function and its capacity as a critical pathway to physical frailty. The network of these keywords effectively maps the conceptual territory where localized oral impairment translates into systemic health risk. The oro-pharyngeal musculature participates in a bidirectional relationship with systemic frailty through the oral sarcopenia paradigm. Atrophy of masticatory and lingual muscles precipitates functional declines in both chewing efficiency and swallowing coordination, while simultaneously contributing to the systemic sarcopenic phenotype through nutritional compromise (31, 32). Frailty-associated systemic inflammation and metabolic dysregulation exacerbate oral muscle degeneration, establishing a self-perpetuating cycle of oro-systemic functional decline. The clinical consequences are particularly severe, with impaired swallowing function (dysphagia) elevating aspiration risk by 3–5-fold in affected older adults, significantly increasing susceptibility to aspiration pneumonia and related complications (33).

### 4.3 Oral health and frailty outcomes: a multidimensional perspective

#### 4.3.1 Cognitive function implications

Bibliometric analysis confirms that cognitive function constitutes an expanding dimension of oral health and frailty research. While keywords such as cognitive impairment occupy a discernible yet non-central position in the co-occurrence network (Figure 7), the pronounced citation burst of “dementia” (2020–2021) (Figure 9), highlights its emergence as a focused research frontier. This trend reflects a conceptual shift within the field toward elucidating oral-systemic-neural connections. Epidemiologic studies consistently show that dentate older adults exhibit superior cognitive performance compared to their edentulous counterparts, with tooth retention demonstrating a dose-dependent protective effect (34, 35). Importantly, frailty status moderates this relationship, with preserved dentition showing stronger cognitive protective effects in prefrail compared to robust individuals (36). The keyword burst of dementia (2020–2021) and the thematic cluster on cognitive impairment suggest a growing research focus on the oral–systemic–neural axis, which may inform future nursing and clinical interventions aimed at integrating oral health into geriatric care protocols (37). While current evidence remains predominantly observational, these findings underscore the need for randomized controlled trials examining cognitive outcomes following oral rehabilitation interventions. Future research should further explore the role of oral health in multidisciplinary collaboration and enhance its integration with frailty management.

#### 4.3.2 Quality of life

Bibliometric analysis substantiates the significant role of quality of life within the oral health-frailty research domain. The keyword “quality of life” not only demonstrates high frequency (Count = 33) (see Supplementary Table 6) but also considerable betweenness centrality (Centrality = 0.20), indicating its function as a critical nexus linking clinical oral health status with psychosocial and functional wellbeing in frailty research. The impact of oral health deterioration extends beyond physiological consequences to profoundly affect psychosocial wellbeing. Compromised masticatory efficiency secondary to tooth loss and periodontal disease frequently precipitates nutritional deficiencies, particularly of high-quality proteins and micronutrients essential for musculoskeletal health (38, 39). This nutritional compromise occurs alongside often-overlooked psychosocial sequelae: dental pain, halitosis, and altered facial esthetics contribute to social withdrawal and dining-related anxiety. The resultant isolation and diminished life satisfaction create a maladaptive cycle wherein reduced social engagement further exacerbates physical inactivity and depressive symptoms (40). These findings highlight the importance of incorporating patient-reported outcome measures in oral health assessments to capture the full spectrum of quality-of-life impacts.

#### 4.3.3 Nutritional mediation pathways

Bibliometric analysis confirms nutrition as a central and persistent theme in oral health-frailty research. The high frequency of “nutritional status” and citation bursts of “malnutrition” and “nutritional status” (2015–2020) (Figure 9), underscore its established role as a key mechanistic pathway. This reflects broad scholarly consensus that oral impairments contribute to frailty largely through nutritional compromise. Nutritional status serves as a critical intermediary in the oral health-frailty relationship, with mounting evidence identifying specific dietary patterns that either mitigate or exacerbate risk. Oral health impairment precipitates a characteristic dietary shift toward softer, highly processed foods with increased refined carbohydrate content and reduced nutrient density (41). Despite recognition of these pathways, the clinical integration of oral-nutritional interventions remains limited, with fewer than 20% of geriatric care programs incorporating coordinated dental and dietary assessments (42). Implementation barriers include inadequate interdisciplinary training and reimbursement challenges, highlighting needs for healthcare system restructuring. The high frequency of nutritional status and citation bursts of malnutrition (2015–2020) underscore nutrition as a central mechanistic pathway in the oral health–frailty relationship. This suggests that future interventions might benefit from integrating oral functional assessments into nutritional evaluations, as reflected in the emerging research themes. This approach not only specifically addresses nutritional deficiencies caused by masticatory and swallowing impairments, but also effectively interrupts the interdependent pathological progression of “oral function decline-malnutrition-frailty exacerbation,” thereby significantly improving overall nutritional status and clinical outcomes in geriatric patients with frailty (43).

#### 4.3.4 Clinical care challenges

The significant presence of the “nursing home” cluster (Figure 8) underscores institutional care as a major focal point for research on implementation barriers. This finding provides empirical support for the documented clinical-practical divide, exemplified by studies showing that while about 60% of long-term care residents have chewing difficulties, fewer than 30% receive regular dental assessments (44, 45). The bibliometric prominence of this theme further reflects persistent systemic challenges cited in the literature, including severe workforce shortages, insufficient staff training, and a widespread underestimation of oral health’s importance in systemic health (46, 47). Therefore, the “nursing home” cluster not only maps a key research area but also bibliometrically corroborates the critical gap between evidence and practice in geriatric oral care, consistent with reports of nursing staff constrained by limited training and resources (48, 49).

### 4.4 Assessment methodologies

The keyword burst analysis (Figure 9) provides direct bibliometric evidence of a methodological shift in the field, marked by strong citation bursts for quantifiable functional parameters such as “masticatory performance” (2018–2020) and “occlusal force” (2018–2020). This trend indicates that the research frontier is actively moving from broad conceptual associations toward the refinement of specific, objective assessment tools to operationalize the diagnosis of “oral frailty.”

This data-driven evolution aligns with recent advances in frailty assessment, which have begun incorporating oral health parameters, reflecting a growing recognition of their prognostic value. Approximately 40% of contemporary frailty indices now include at least one oral health component, typically evaluating the very functions highlighted by the burst keywords (3, 50–52). Parallel developments in oral health assessment tools, such as the Geriatric Oral Health Assessment Index (GOHAI), now incorporate frailty-relevant domains including nutritional intake and social functioning (53).

However, the bibliometric landscape, characterized by the sequential emergence of distinct measurement concepts, also underscores persistent standardization challenges. The heterogeneity in measurement approaches limits cross-study comparability, an issue compounded by the fact that only a minority of existing instruments have undergone rigorous validation in frail populations, indicating a clear need for future methodological refinement (53).

## 5 Future directions and study limitations

The scientific community is demonstrating a rapidly growing interest in the relationship between oral health and frailty (54, 55). As evidenced by the publication trends analyzed in this study, annual output has exhibited exponential growth since 2020, accounting for over 60% of the total publications. This firmly establishes oral health and frailty as a rapidly emerging frontier within geriatric research. Priority research directions should include: (1) Mechanistic studies elucidating oral microbiome-neuroimmune interactions; (2) Comparative effectiveness trials of integrated oral health-nutrition-exercise interventions; and (3) Development of culturally adapted assessment tools for diverse populations. Such advances will require unprecedented interdisciplinary collaboration between dental professionals, geriatricians, and public health researchers to translate the growing mechanistic understanding into clinical practice. While our bibliometric approach provides novel insights into research trends, several limitations warrant consideration. Database constraints limited our analysis to the Web of Science (WoS) Core Collection, potentially excluding relevant studies indexed elsewhere. Although this database is widely used in bibliometrics for its high-quality, standardized data, this reliance means that some pertinent literature from other databases (e.g., Scopus, PubMed) or regional journals may not have been captured. This is a recognized constraint of single-database bibliometric studies and may influence the comprehensiveness of the mapped literature landscape. However, WoS’s rigorous journal selection process and comprehensive citation tracking ensure robust identification of core literature (15). Future studies could address this limitation by developing robust methods to integrate and harmonize data from multiple sources, thereby capturing a more comprehensive view of the field.

## 6 Conclusion and clinical implications

This bibliometric analysis delineates a rapidly evolving research landscape on oral health and frailty, characterized by distinct intellectual structures and collaborative patterns. Key bibliometric trends reveal that Japan, China, and the United States are the leading contributors, with Japan dominating both publication output and citation impact. Influential journals such as Gerodontology and the Journal of the American Geriatrics Society have served as central platforms for knowledge dissemination, while authors like Hirohiko Hirano have played pivotal roles in shaping the field’s conceptual and methodological foundations.

Keyword co-occurrence and burst analyses further highlight the evolution of research fronts: from foundational concepts such as “tooth loss” and “oral frailty,” to emerging themes including “oral sarcopenia,” “dementia,” “nutritional status,” and “periodontitis.” These trends reflect a progressive refinement from epidemiological association to mechanistic exploration and interventional research.

Despite a mature knowledge base substantiating the oral–frailty relationship, the field faces persistent challenges, including fragmented international collaboration, limited standardization of assessment tools, and insufficient clinical integration. To address these gaps and further advance this interdisciplinary field, future research should foster international collaboration and integrate standardized frailty assessment frameworks into oral health studies. Such efforts will be crucial for developing culturally adapted instruments, conducting interdisciplinary randomized trials, and implementing integrated care models that synergize oral, nutritional, and physical interventions. By fostering global partnerships and implementing person-centered, preventive strategies, healthcare systems can better address the oral–systemic health continuum and promote healthy aging across diverse populations.

## Data Availability

The original contributions presented in this study are included in this article/Supplementary material, further inquiries can be directed to the corresponding author.
