# Supplementary material for: Research trends in oral health and frailty studies: a bibliometric and visual analysis
Source: Front Med (Lausanne). 2026 Jan 2;12:1610582. doi: 10.3389/fmed.2025.1610582 (PMC12808489; doi:10.3389/fmed.2025.1610582)
Supplement: Supplementary file 1 [file Data_Sheet_1.docx]

***Table 1.*** *The top 10 countries/regions in terms of publication volume.*

| **Rank** | **Countries** | **Count** | **Centrality** | **Citations** |
| --- | --- | --- | --- | --- |
| **1** | JAPAN | 83 | 0.22 | 2295 |
| **2** | PEOPLES CHINA | 36 | 0.09 | 198 |
| **3** | USA | 30 | 0.46 | 465 |
| **4** | ENGLAND | 25 | 0.21 | 762 |
| **5** | NETHERLANDS | 22 | 0.02 | 820 |
| **6** | SOUTH KOREA | 16 | 0.00 | 81 |
| **7** | SWITZERLAND | 11 | 0.14 | 205 |
| **8** | BRAZIL | 11 | 0.08 | 234 |
| **9** | CANADA | 11 | 0.03 | 119 |
| **10** | AUSTRALIA | 10 | 0.01 | 137 |

***Table 2.*** *The top 10 institutions in terms of publication volume.*

| **Rank** | **institution** | **publications** | **Rank** | **Institution** | **Centrality** |
| --- | --- | --- | --- | --- | --- |
| **1** | University of  London | 17 | **1** | Newcastle University - UK | 0.19 |
| **2** | Tokyo Metropolitan Institute of Gerontology | 14 | **2** | University of Adelaide | 0.18 |
| **3** | University of Tokyo | 13 | **3** | Osaka University | 0.16 |
| **4** | National Center for Geriatrics & Gerontology | 12 | **4** | Ghent University | 0.15 |
| **5** | Osaka University | 12 | **5** | University College London | 0.14 |
| **6** | Hokkaido University | 11 | **6** | University of London | 0.09 |
| **7** | King's College London | 9 | **7** | National Center for Geriatrics & Gerontology | 0.09 |
| **8** | Niigata University | 7 | **8** | Niigata University | 0.05 |
| **9** | Academic Center for Dentistry Amsterdam | 7 | **9** | Chung Shan Medical University | 0.05 |
| **10** | Ghent University | 6 | **10** | Academic Center for Dentistry Amsterdam | 0.04 |

***Table 3.*** *The top 10 authors and co-authors.*

| **Rank** | **Author** | **publications** | **Centrality** | **HalfLife** | **Citations** | **Co-cited author** | **Citations** |
| --- | --- | --- | --- | --- | --- | --- | --- |
| **1** | Hirano, Hirohiko | 10 | 0.02 | 4.5 | 1228 | FRIED LP | 105 |
| **2** | Arai, Hidenori | 7 | 0.01 | 6.5 | 330 | TANAKA T | 81 |
| **3** | Iijima, Katsuya | 7 | 0.01 | 0.5 | 658 | IWASAKI M | 80 |
| **4** | Shirobe, Maki | 7 | 0 | 0.5 | 410 | WATANABE Y | 77 |
| **5** | Iwasaki, Masanori | 6 | 0 | 3.5 | 352 | CASTREJÓN-PÉREZ RC | 72 |
| **6** | Watanabe, Yutaka | 6 | 0 | 3.5 | 1143 | HAKEEM FF | 64 |
| **7** | Edahiro, Ayako | 5 | 0.01 | 0.5 | 489 | DIBELLO V | 45 |
| **8** | Akishita, Masahiro | 4 | 0.01 | 3.5 | 485 | RAMSAY SE | 41 |
| **9** | Bernabe, Eduardo | 4 | 0 | 3.5 | 389 | MINAKUCHI S | 37 |
| **10** | Ikebe, Kazunori | 4 | 0.01 | 4.5 | 332 | PETERSEN PE | 34 |

***Table 4.*** *The top 10 journals.*

| **Rank** | **Journal** | **publications** | **Citations** | **IF and JCR**  **(2024)** |
| --- | --- | --- | --- | --- |
| 1 | GERODONTOLOGY | 20 | 679 | 2.0,Q2 |
| 2 | BMC GERIATRICS | 19 | 244 | 3.4,Q2 |
| 3 | JOURNAL OF NUTRITION HEALTH AGING | 17 | 344 | 4.3,Q1 |
| 4 | ARCHIVES OF GERONTOLOGY AND GERIATRICS | 15 | 250 | 3.5,Q2 |
| 5 | BMC ORAL HEALTH | 15 | 178 | 2.6,Q1 |
| 6 | GERIATRICS GERONTOLOGY  INTERNATIONAL | 13 | 355 | 2.4,Q2 |
| 7 | INTERNATIONAL JOURNAL OF ENVIRONMENTAL RESEARCH ANDPUBLIC HEALTH | 13 | 168 | 4.6,Q2 |
| 8 | EUROPEAN GERIATRIC MEDICINE | 12 | 72 | 3.5,Q2 |
| 9 | JOURNAL OF ORAL REHABILITATION | 11 | 150 | 3.1,Q1 |
| 10 | JOURNAL OF THE AMERICANGERIATRICS SOCIETY | 8 | 568 | 6.1,Q1 |

***Table 5.*** *The top 10 cited journals.*

| **Rank** | **Co-citations** | **First author** | **Journal** | **Year** | **DOI** | **Centrality** | **IF and JCR**  **(2024)** |
| --- | --- | --- | --- | --- | --- | --- | --- |
| 1 | 48 | Hakeem FF | GERODONTO-LOGY | 2019 | 10.1111/ger12406 | 0.00 | 2.0,Q2 |
| 2 | 46 | Tanaka T | J Gerontol A Biol Sci Med Sci | 2018 | 10.1093/gerona/glx225 | 0.05 | 4.3,Q1 |
| 3 | 42 | Watanabe Y | J AM GERIATR SOC | 2017 | 10.1111/jgs.14355 | 0.15 | 4.3,Q1 |
| 4 | 37 | Dibello V | LANCET HEALTH LONGEV | 2021 | 10.1016/S2666-7568(21)00143-4 | 0.01 | 13.4,Q1 |
| 5 | 33 | Ramsay SE | J AM GERIATR SOC | 2018 | 10.1111/jgs.15175 | 0.19 | 4.3,Q1 |

***Table 6.*** *The top 5 Co-citation.*

| **Rank** | **Co-citations** | **First author** | **Journal** | **Year** | **DOI** | **Centrality** | **IF and JCR**  **(2024)** |
| --- | --- | --- | --- | --- | --- | --- | --- |
| 1 | 48 | Hakeem FF | GERODONTOLOGY | 2019 | 10.1111/ger12406 | 0.00 | 2.0,Q2 |
| 2 | 46 | Tanaka T | J Gerontol A Biol Sci Med Sci | 2018 | 10.1093/gerona/glx225 | 0.05 | 4.3,Q1 |
| 3 | 42 | Watanabe Y | J AM GERIATR SOC | 2017 | 10.1111/jgs.14355 | 0.15 | 4.3,Q1 |
| 4 | 37 | Dibello V | LANCET HEALTH LONGEV | 2021 | 10.1016/S2666-7568(21)00143-4 | 0.01 | 13.4,Q1 |
| 5 | 33 | Ramsay SE | J AM GERIATR SOC | 2018 | 10.1111/jgs.15175 | 0.19 | 4.3,Q1 |

***Table 7.*** *The top 20 keywords.*

| **Rank** | **Counts** | **Keywords** | **Centrality** | **Rank** | **Counts** | **Keywords** | **Centrality** |
| --- | --- | --- | --- | --- | --- | --- | --- |
| **1** | 123 | oral health | 0.42 | **11** | 24 | population | 0.03 |
| **2** | 60 | older adults | 0.05 | **12** | 22 | care | 0.15 |
| **3** | 53 | health | 0.41 | **13** | 22 | oral frailty | 0.03 |
| **4** | 47 | association | 0.36 | **14** | 21 | physical frailty | 0.04 |
| **5** | 47 | adults | 0.12 | **15** | 20 | mortality | 0.28 |
| **6** | 44 | tooth loss | 0.6 | **16** | 20 | older people | 0.06 |
| **7** | 35 | people | 0.22 | **17** | 19 | disease | 0.1 |
| **8** | 33 | quality of life | 0.2 | **18** | 19 | risk | 0.05 |
| **9** | 27 | prevalence | 0.05 | **19** | 19 | oral function | 0.05 |
| **10** | 26 | frailty | 0.04 | **20** | 17 | age | 0.13 |
