# Supplementary material for: Research trends in oral health and frailty studies: a bibliometric and visual analysis
Source: Front Med (Lausanne). 2026 Jan 2;12:1610582. doi: 10.3389/fmed.2025.1610582 (PMC12808489; doi:10.3389/fmed.2025.1610582)
Supplement: Supplementary file 2 [file Supplementary_file_1.docx]

| **Component** | **Description** |
| --- | --- |
| **Database** | Web of Science Core Collection |
| **Search Date** | 16 January 2025 |
| **Years** | 2000–2024 |
| **Document Types** | Article OR Review |
| **Language** | English |
| **Boolean Query** | TS=("Oral health" OR "Mouth Rehabilitation" OR "Dental Clinics" OR "Dental Health Surveys" OR "Mouth Diseases" OR "Oral frail*" OR "Mouth health" OR "Dentistry" OR "Oral hygiene" OR "Dental hygiene" OR "Mouth hygiene" OR "Teeth health" OR "Teeth hygiene" OR "Dental health") AND TS=("Frailties" OR "Frailness" OR "Frailty Syndrome" OR "debilitation" OR "Frail") |
