# Supplementary material for: Research trends in oral health and frailty studies: a bibliometric and visual analysis
Source: Front Med (Lausanne). 2026 Jan 2;12:1610582. doi: 10.3389/fmed.2025.1610582 (PMC12808489; doi:10.3389/fmed.2025.1610582)
Supplement: Supplementary file 3 [file Supplementary_file_2.docx]

| **Note Types** | **Time Slicing (Years per slice)** | **Selection Criteria** | | **Prunig** |
| --- | --- | --- | --- | --- |
|  |  | **g-index** | **Top-N** |  |
| **Country** | 2000-2024 (1) | **25** | **50** | **Pathfinder** |
| **Institution** | 2000-2024 (1) | **25** | **50** | **Pathfinder** |
| **Author** | 2000-2024 (1) | **16** | **50** | **Pathfinder** |
| **Cited journal** | 2000-2024 (1) | **16** | **50** | **Pathfinder** |
| **Co-citation** | 2000-2024 (1) | **25** | **50** | **Pathfinder** |
| **keywords** | 2000-2024 (1) | **16** | **50** | **Pathfinder** |
